# Morbid History and Dissection of a Young Woman Who Died of a Palsy of the Heart within Three Hours of Her Being Taken Ill

**Published:** 1817-09

**Authors:** L. A. Golis

**Affiliations:** of Vienna.


*For the London Medical and Physical Journal.*

*Morbid History and Dissection of a young Woman who died of a Palsy of the Heart within three hours of her being taken ill;* by Dr. L. A. GOLIS, of Vienna. Communicated by Dr. VON EMBDEN.

MRS. T. Z., twenty-five years of age, a lusty round little woman, of a good habit of body, lively and serene temper, mother of a boy three years old, and suckling a fine infant of four months, a few days previous to her last complaint, complained of orgasm and a burning heat in the chest, feeling, in the centre of it, as she expressed herself, a sensation as from a heated oven, spreading its glow in all directions: besides this, she was much troubled with a dry cough. Having neither pain, cough, nor fever of any consequence, she took, without asking advice, infusions of marsh-mallow and mucilaginous soups. On the 6th of August, 1815, about eleven o'clock in the night, being still up up with the rest of the family, sprightly and brisk, they rallied her on account of a swelling in her thyroid gland, that had remained after her first lying-in, and increased in the last, from which they jokingly presaged her a goitre. But this accident had in reality been very troublesome to this otherwise healthy young woman, in ascending hills or stairs during her second pregnancy, by causing frequent palpitations, and greater pulsations through the whole body, and rendering respiration very difficult. About half-past eleven o'clock, she suckled her babe, kissing and toying with it, and, having lulled it to sleep, lay down herself, and slept full four hours. About four o'clock in the morning, she suddenly awoke with anxiety and dread of suffocation, and, jumping out of the bed instantly, she ran into the next room, and forced open the window, to fetch breath. Her husband, roused by the noise caused by her suddenly leaping out of the bed, and, by the glimmer of a night-taper, perceiving her to run to the window, quickly hastened to her assistance; and her brother, of whose apartment she was opening the window, also terrified by the noise and the sight of a white figure, which he discovered to be his sister, hastened likewise to assist her. Her husband and brother, being of opinion she intended to throw herself out of the window of a fourth floor, directly pulled her away from the window, when speechless and senseless she sunk into their arms, her hands and feet cold: they led her to an arm-chair, and instantly sent for me. Before half an hour had elapsed, I was at the side of the unhappy patient, who was unable to answer either by words or looks, and only discovered her little remaining life by a rattling respiration. During the few minutes I observed her, and whilst hearing the particulars, she seemed to recover her senses, and uttered a few scarcely intelligible words. Her face, hands, and legs were swoln and blue, cold, and covered with a death-like sweat; the pulse small, weak, soft, not very feverish, and had only an arterious movement; and no beating of the heart was perceivable during the remissions of the apparent spasmodic symptoms, in any direction or situation of the body whatsoever. Respiration was difficult: hardly five rattling respirations succeeded each other in two minutes. The breath was very warm, and the patient kept her mouth open even during the remission. The tongue was of a bluish red. During and after the asthmatic fit, saliva flowed from the mouth. She complained of violent thirst; could, however, but with difficulty swallow any liquid, but suffered the most part to run out of her mouth, and a rattling was heard in her throat on swallowing. The eye was in the natural

 state, state, the point of her nose white, and the alæ nasi at every inspiration approached the septum; the lips, the points of the fingers and toes, particularly the nails, livid. She did not complain of any pains, but of great debility, and an indescribable fear of suffocation, of which latter she felt momentary relief by breaking wind upwards.

This sudden attack after midnight, the remission of the symptoms, the great irritability of the nerves of this subject even in health, the eructations, and the great involuntary discharge of urine during the first asthmatic paroxysm, and during the remission,—all this together, prompted me to consider the affection as spasmodic, and thus to institute a treatment I had found indicated and efficacious in similar cases. The back was instantly covered with a large blister from the neck down to the loins, and an infusion of valerian, the emulsio arabica, and two grains of musk, prescribed to be given every two hours. The patient was ordered to be put to bed, and the extremities to be warmed with dry fomentations; but these orders were scarcely executed, and the medicines offered to the patient, when suddenly a second attack took place, and she died of suffocation after a remission of half an hour, and three hours of dreadful sufferings, without having taken one grain of the medicines prescribed for her.

*Dissection.*—Forty-eight hours after the decease, the surgeon, Mr. Trirnhofer, undertook this task. After dissecting the common integuments, and all the fat that gave the deceased such a charming plump appearance, the thyroid gland appeared almost thrice as large as natural. The trachea, pressed by the gland morbidly enlarged, was contracted from the front towards the back, and dilated on both sides. The sternum was found entirely deviating from its just shape: its anterior and posterior surface was full of protuberances and depressions, and the ensiform cartilage not broader than the two edges to which the costal cartilages are joined, which gave it more the appearance of a large quadralateral coarse file than that of a sword. Similarly deviating from the natural shape was the structure of the costal cartilages, which were thick, crooked, and distorted. The ribs deviated so much from their proper direction as to form a small protuberance on the right side, which had, however, been completely hidden by the fat and her well-contrived garments. There was no water in the pectoral cavity; the lungs, which in several places adhered to the pleura, were flaccid, but for the rest healthy. The distended pericardium contained four ounces of water, and the heart more resembled a thick membranaceous flaccid bag filled with blood

2 than than a muscular body; its external circumference, its shape, and size, proved the morbid state of this important organ. The atria, in particular the right one, was dilated to a monstrous degree, but the ventricles and their interior structure regularly formed. The vena cava and the pulmonary veins were more turgid with venous blood than the heart itself. We found no adhesions of the pericardium by pseudo membranes, polypous concretions, or ossifications, and the situation of that organ was also quite natural. From these appearances, it was, indeed, easy to explain the complaints during life, as also the asthmatic fits, but by no means that instantaneous total paralysis of the heart, that rapid suffering wh ch proved fatal in the space of three hours. The aorta and the pulmonary vein were divided from the heart. On dividing the latter, we found its interior surface slightly inflamed, but the ascendant aorta, with all its ramifications, down to the smallest of them, inflamed in the highest degree, and some places overspread with dark-red spots. The blood contained in them was decomposed in serum, cruor, and lymph, imitating the form of a long worm of various thickness. The abdominal and pelvic viscera were quite natural; only the vena portarum was overcharged with blood. The cranial and spinal cavities offered nothing morbid, except that the blood-vessels were turgescent with blood, as a natural consequence of the morbid circulation.

The principal symptoms lasting but three hours, with short but plain remissions, justified my method of treatment, though not the inflammatory appearances in the arterial system. Had I been called in earlier, when the patient complained of great heat in the cavity of the breast, flushings, and dry cough, the inflammation of the vessels, to which the great Peter Frank first directed my attention, and with which a manifold experience made me better acquainted, would not have been mistaken or overlooked by me; and perhaps a vigorous anti-inflammatory proceeding, which has rendered me the most essential services with other patients of this class, might also have saved this young woman, or at least prevented her sudden death. But at that moment when I first saw the patient, not guided by any recollection, and in presence of the above symptoms, what physician would not have adopted the same treatment?

